# Unlocking the hidden variation from wild repository for accelerating genetic gain in legumes

**DOI:** 10.3389/fpls.2022.1035878

**Published:** 2022-11-09

**Authors:** Gurjeet Singh, Santosh Gudi, Amandeep, Priyanka Upadhyay, Pooja Kanwar Shekhawat, Gyanisha Nayak, Lakshay Goyal, Deepak Kumar, Pradeep Kumar, Akashdeep Kamboj, Antra Thada, Shweta Shekhar, Ganesh Kumar Koli, Meghana DP, Priyanka Halladakeri, Rajvir Kaur, Sumit Kumar, Pawan Saini, Inderjit Singh, Habiburahman Ayoubi

**Affiliations:** ^1^ Department of Plant Breeding and Genetics, Punjab Agricultural University, Ludhiana, Punjab, India; ^2^ Division of Crop Improvement, Plant Breeding and Genetics, Indian Council of Agricultural Research (ICAR)-Central Soil Salinity Research Institute, Karnal, Haryana, India; ^3^ Department of Plant Breeding and Genetics, Sri Karan Narendra Agriculture University, Jobner, Rajasthan, India; ^4^ Department of Genetics and Plant Breeding, Indira Gandhi Krishi Vishwavidyalaya, Raipur, Chhattisgarh, India; ^5^ Department of Genetics and Plant Breeding, Chaudhary Charan Singh Haryana Agricultural University, Hisar, Haryana, India; ^6^ Department of Plant Molecular Biology and Biotechnology, Indira Gandhi Krishi Vishwavidyalaya, Raipur, Chhattisgarh, India; ^7^ Department of Genetics and Plant Breeding, Anand Agricultural University, Anand, Gujarat, India; ^8^ Department of Agronomy, Punjab Agricultural University, Ludhiana, Punjab, India; ^9^ CSB-Central Sericultural Research & Training Institute (CSR&TI), Ministry of Textiles, Govt. of India, Jammu- Kashmir, Pampore, India

**Keywords:** legume improvement, gene pool, introgression, pre-breeding, wild species

## Abstract

The fluctuating climates, rising human population, and deteriorating arable lands necessitate sustainable crops to fulfil global food requirements. In the countryside, legumes with intriguing but enigmatic nitrogen-fixing abilities and thriving in harsh climatic conditions promise future food security. However, breaking the yield plateau and achieving higher genetic gain are the unsolved problems of legume improvement. Present study gives emphasis on 15 important legume crops, i.e., chickpea, pigeonpea, soybean, groundnut, lentil, common bean, faba bean, cowpea, lupin, pea, green gram, back gram, horse gram, moth bean, rice bean, and some forage legumes. We have given an overview of the world and India’s area, production, and productivity trends for all legume crops from 1961 to 2020. Our review article investigates the importance of gene pools and wild relatives in broadening the genetic base of legumes through pre-breeding and alien gene introgression. We have also discussed the importance of integrating genomics, phenomics, speed breeding, genetic engineering and genome editing tools in legume improvement programmes. Overall, legume breeding may undergo a paradigm shift once genomics and conventional breeding are integrated in the near future.

## Introduction

Rising human population, fluctuating climates, and depleting arable land, coupled with lower productivity and post-harvest losses, pose a serious threat to global food security. Furthermore, surging pests and diseases challenge global researchers to develop stress resilient, high-yielding, nutritious crops to alleviate hunger and deprivation (Bakala et al., 2020; Saini et al., 2020). Legumes belong to the “Fabaceae” family, which is the third largest family of angiosperms and is the second most important family of agriculture crops following cereals, with approximately 800 genera and 20,000 species (Smýkal and Konečná, 2014). Legumes are an integral component of the human food because they provide low-cost, nutrient-rich proteins, vitamins, and minerals, and also aid in preventing chronic diseases (Arnoldi et al., 2015; Vaz Patto et al., 2015). Among the legume crops, grain legumes contribute nearly 27 per cent of the world food production and also act as the single largest source of vegetable protein (~33%) in the human diet (Young and Bharti, 2012).

**Figure 1:**
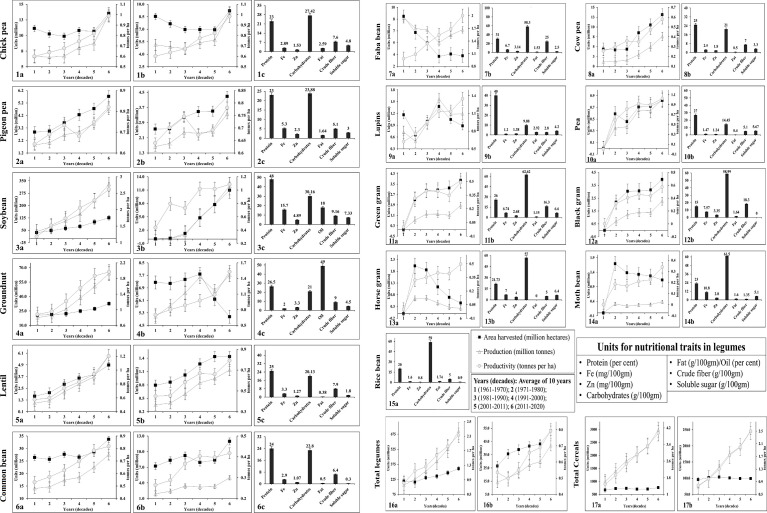
Area (in million hectare), production (in million tonnes), and productivity (in tonnes per hectare) from 1961 to 2020 (World and India) and nutritional quality traits (protein, Fe, Zn, carbohydrates, fat/oil, crude fiber, and soluble sugar) of legume crops. Area, production, productivity trend of World and India and nutritional quality traits (right) of chickpea (1a-c); pigeon pea (2a-c); soybean (3a-c), groundnut (4a-c), lentil (5a-c), and common bean (6a-c). World’s area, production, productivity and nutritional quality traits of faba bean (7a-b), cowpea (8a-b), and lupins (9a-b); India’s area, production, productivity and nutritional quality traits of pea (10a-b), green gram (11a-b), black gram (12a-b), horse gram (13a-b), and moth bean (14a-b); nutritional quality traits of rice bean (15a); Area, production, and productivity of total legume crops in world (16a) and India (16b); Area, production, and productivity of total cereals (including millets) in world (17a) and India (17b). Information for the worlds and India’s area, production and productivity (from 1961 to 2020) of all crops were collected from FAOSTAT (https://www.fao.org) and Indiastat (https://www.indiastat.com) respectively. Information for the nutritional quality traits were collected from the published articles of respective crops.

Legumes have the capability to fix atmospheric nitrogen and are also believed to be involved in carbon sequestration and soil amelioration. Hence, they are considered an essential feature of sustainable agricultural production, particularly in arid regions (Duc et al., 2015). Their ability to improve soil fertility by establishing symbiotic associations with nitrogen (N)-fixing bacteria and phosphorus (P)-absorbing arbuscular mycorrhizal fungi has been rewarded since antiquity, and they are considered a cost-effective and resource-saving alternative to inorganic fertilisers. Furthermore, legume crops demand less water for growth and development and can also withstand extreme climatic conditions (Peix et al., 2015). Nutritionally rich, climate-resilient, less resource-demanding legume crops have attracted the interest of researchers and farmers to grow more legumes, which has resulted in increased area and production of these crops over the last six decades (**Figure 1**). Furthermore, because of their short duration, they also facilitate intercropping or crop rotation with cereal crops and thereby increases the farm income (Araujo et al., 2015).

In addition, the simple genetic architecture of legume crops also contributed to the field of genetics. Among the crop species, garden pea (*Pisum sativum* L.), was the first experimental crop used by Gregor Johann Mendel (Father of Genetics) in his pioneering work to understand the basics of heredity and variation, which paved the way for establishing new branch of biological science, i.e., *Genetics* (Smýkal and Konečná, 2014). Later, significant progress has been made in the breeding programme for improving annual and perennial legumes (Annicchiarico et al., 2015; Boelt et al., 2015). Furthermore, with the advent of molecular biology and high-throughput sequencing technology, genome sequencing has become cheaper and the genome sequences of most of the legume crops are now available. Genome sequences revealed that legume crops differ significantly in their genome size, basic chromosome number, ploidy level, and reproductive biology, despite their close relatedness (**Table 1**). Researchers can exploit available genome information to make genetic improvements to legume crops.

**Table 1:** List of legume crops with their genomic information.

S. No.	Crops	Botanical name	2n	Genome size (Mb)	Number of genes	Parents used for sequencing	Method used for sequencing	Reference
1.	Chickpea	*Cicer arietinum* L.	16	738.09	28,269	CDC Frontier	Whole genome shotgun	(Varshney et al., 2013c)
2.	Pigeonpea	*Cajanus cajan* (L.) Millsp.	22	833.07	48,680	ICPL 87119 (known as Asha)	Illumina next-generation sequencing platform	(Varshney et al., 2012a)
3.	Soybean	*Glycine max* (L.) Merr.	40	1,150	46,430	Williams 82	Whole genome shotgun	(Schmutz et al., 2010)
4.	Groundnut	*Arachis hypogaea* L.	40	2,540	83,709	Shitouqi (zh.h0235, known Chinese cultivar)	Illumina	(Zhuang et al., 2019)
5.	Lentil	*Lens culinaris* Medikus	14	4,300	–	CDC Redberry	Restriction site associated DNA, genotyping-by-sequencing approach	(Kumar et al., 2021)
6.	Common bean	*Phaseolus vulgaris* L.	22	587	27,197	G19833	whole-genome shotgun sequencing strategy	(Schmutz et al., 2014)
7.	Faba bean	*Vicia faba* L.	12	13,000	–	Hedin 2	PacBio CLR	(Carrillo-Perdomo et al., 2020)
8.	Cowpea	*Vigna unguiculata* (L.) Walp.	22	640.6	29,773	IT97K-499-35	PacBio single-molecule real-time sequencing	(Lonardi et al., 2019)
9.	Lupin	*Lupinus angustifolius* L.	32-52	609	33,076	Tanjil	Illumina	(Hane et al., 2017)
10.	Pea	*Pisum sativum* L.	14	4,450	44,756	Caméor	Whole genome sequencing	(Kreplak et al., 2019)
11.	Green gram	*Vigna radiata* (L.) R. Wilczek	22	579	22,427	VC1973A	Whole genome sequencing	(Kang et al., 2014)
12.	Black gram	*Vigna mungo* (L.) Hepper	22	475	42,115	Hepper	Whole genome sequencing	(Jegadeesan et al., 2021)
13.	Horse gram	*Macrotyloma uniflorum* (Lam.) Verdc.	20	279.12	36,105	HPK-4	IlluminaHiSeq 2000	(Shirasawa et al., 2021)
14.	Moth bean	*Vigna aconitifolia* (Jacq.) Marechal	22	416	–	–	–	(Yundaeng et al., 2019)
15.	Rice bean	*Vigna umbellata* Thunb.	22	414	31,276	VRB3	Illumina and PacBio platforms	(Kaul et al., 2019)

Despite their economic importance and health benefits, the rate of genetic gain achieved in legume crops (viz., 27.4 kg/ha/year) over last two decades has been very slow compared to that achieved in cereals (viz., 51.5 kg/ha/year) (**Figure 1**). A comprehensive study on a soybean historical dataset of 80 years revealed a genetic gain of 26.5 kg/ha/year. This gain was associated with increased light interception, energy conversion, and partitioning efficiency of improved soybean lines (Koester et al., 2014). Furthermore, over the last two decades, the rate of genetic improvement in chickpea is 0.24 tonnes/ha/year, pigeonpea is 0.14 tonnes/ha/year, ground nut is 0.4 tonnes/ha/year, soybean is 0.56 tonnes/ha/year, common bean is 0.12 tonnes/ha/year, lentil is 0.37 tonnes/ha/year, faba bean is 0.46 tonnes/ha/year, lupin is 0.21 tonnes/ha/year, and cowpea is 0.23 tonnes/ha/year (**Figure 1**). The slow genetic gain achieved in legume crops is associated with their narrow genetic base and lack of innovative breeding tools to introgress desirable genomic regions for several biotic (causing 15-100% yield loss) and abiotic (causing 8-86% yield loss) stresses from wild germplasms into cultivars (Araujo et al., 2015). The following factors are considered as the primary barriers which limit the genetic gains in legume crops: (i) a narrow genetic base due to the accumulation of domestication syndrome traits; (ii) monophyletic evolution (in contrast to multi-event evolution in wheat and brassica); (iii) recurrent use of the same breeding lines in legume improvement; and (iv) un-exploitation of wild resources. Difficulty in crossing and lack of high-throughput phenotyping facilities will further reduces the potential of exploiting the rich sources of genetic diversity present in the secondary and tertiary gene pools (Singh et al., 2013b).

In addition, indeterminacy, plant morphology, environmental sensitivity, slow growth rate, and lack of management practises and government policies will further hinder legume improvement. Indeterminacy leads to non-synchronous flowering and maturity, which will affect the crossing and mechanical harvesting, respectively. Legume crops have poor photosynthetic efficiency, slow dry matter accumulation, reduced seedling vigour and canopy development, poor source-sink relationship, and rapid leaf senescence. Furthermore, they have reduced leaf area index (0.7-2.0) compared to cereal crops (3-6), which will reduce the gas exchange and photosynthetic efficiency. Legumes produce many flowers, but due to a lack of significant nutrient assimilation, most of them will fall before setting into fruit. For instance, about 70-90% and 80-95% of the produced flowers will drop in green gram and pigeon pea, respectively (Alam Mondal et al., 2011). Legume crops are also sensitive to high temperatures, photoperiod, and genotype × environment interaction. Furthermore, slow growth rates during early developmental stages result in a dense weed infestation which competes for nutrients, water, light, and space. Weeds will also act as alternative hosts for many pests and diseases. Poor management practices, including seed replacement, rainfed farming, and post-harvest losses, are also responsible for the reduced productivity of legume crops. Government policies, including lack of guaranteed markets, minimum support prices, storage facilities, crop insurance, and processing industry, are all factors that have been overlooked when it comes to legume crops.

Given the importance of legume crops in a sustainable agricultural system and food security, we prepared a comprehensive review on challenges and strategies to overcome these challenges in legume breeding programmes. Furthermore, we discussed the advantages of integrating advanced genomics and phenomics approaches into distant hybridization to accelerate alien gene introgression and achieve higher genetic gain in these crops.

## Theory of gene pool

**Figure 2:**
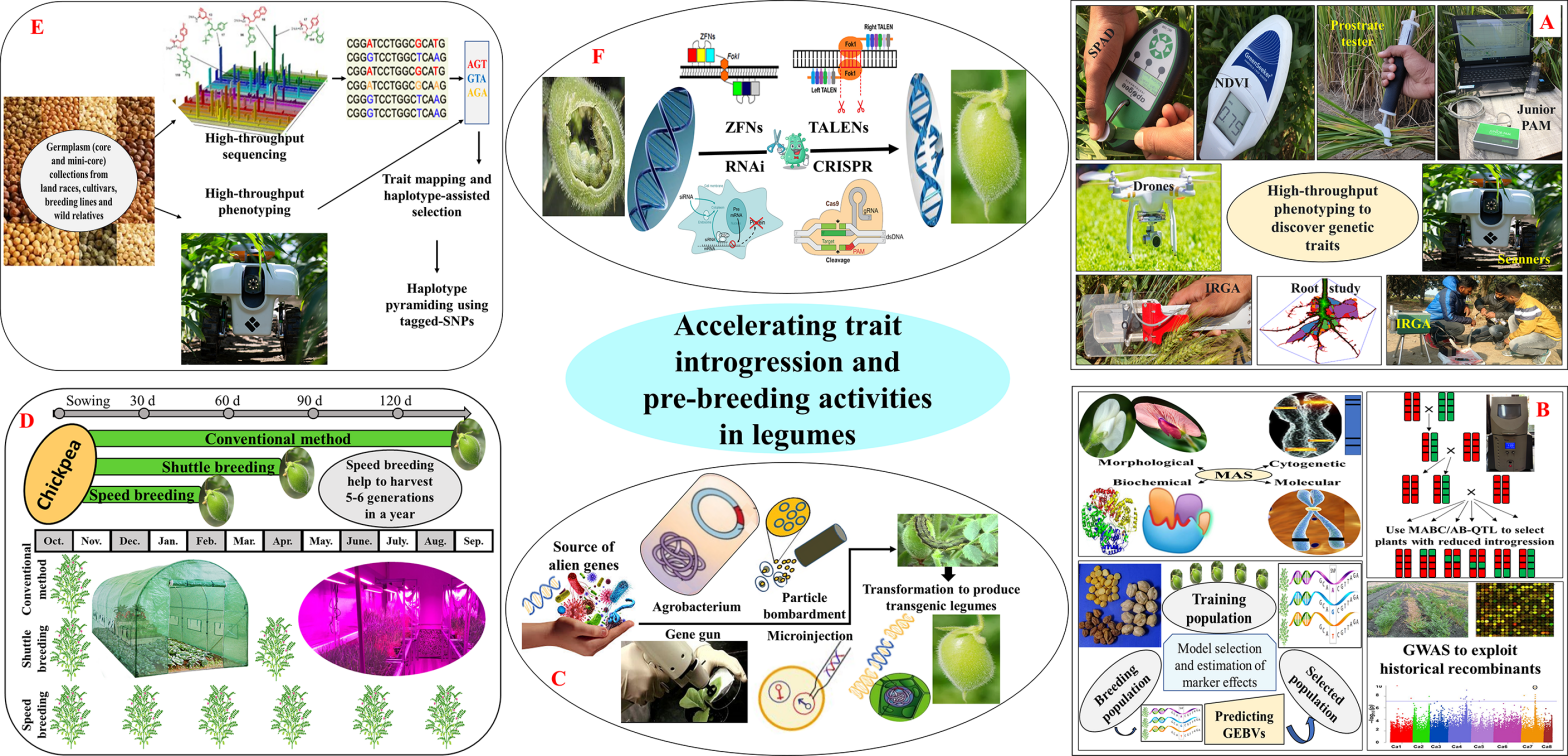
Integrated approaches for accelerating trait introgression and achieving higher genetic gain in legumes; **(A)** high-throughput phenotyping (SPAD-meter, green seeker, prostrate tester, junior PAM, drones, scanners, IRGA, etc.) to take full advantage of large-scale genomic data sets built by next-generation sequencing (NGS) tools; **(B)** bringing genomic tools like, marker-assisted selection (MAS), marker-assisted backcrossing (MABC), advanced backcross quantitative trait loci mapping (AB-QTL), marker-assisted recurrent selection (MARS), genomic selection (GS), and genome wide association studies (GWAS) to identify and introgress desirable genes or QTLs into legumes with high efficacy; **(C)** biotechnology (gene gun, particle bombardment, microinjection, and *Agrobacterium*) mediated transformation of alien genes to produce pest, disease and herbicide resilient transgenic crops; **(D)** dialling physiology of plants with protracted photoperiod, elevated temperature and CO_2_ coupled with immature seed harvest to accelerate generation advancement; **(E)** developing tailor-made crop varieties by using haplotype-based breeding; **(F)** targeted genome editing using meganucleases, zinc finger nucleases (ZFNs), transcription activator-like effector nucleases (TALENs), and clustered Regularly Interspaced Short Palindromic Repeats (CRISPRs) to produce non-transgenic, genome edited crops.

Gene pool (GP) is a relationship among crop plants and related taxa that could be useful to breeders for crop improvement (Harlan and de Wet, 1971). Based on ease of hybridization, GPs can be classified as; primary (GP-1), secondary (GP-2), and tertiary (GP-3). GP-1 includes all germplasm lines (viz., cultivars, land races, and elite germplasm lines) which freely hybridized and produce fully viable offsprings. GP-2 includes all wild relatives of the crop species which can be crossed with GP-1, but with great difficulty. Gene transfer from GP-2 to cultivars is possible but may be challenging. GP-3 is the outer limit of potential genetic resources associated with cultivated species. GP-3 includes all the wild species of crop plants, which cannot produce fertile hybrids upon hybridization with members from GP-1. Germplasm resources available in GP-2 and GP-3 help in broadening the genetic diversity of GP-1 through distant hybridization. However, there are several pre- and post-fertilization barriers which limits introgression of desirable traits from GP-2 or Gp-3 to Gp-1 (**Figure 2**). However, by implementing different strategies, such as delayed and bud pollination, mentor pollination, embryo rescue, ploidy adjustment, bridging species, somatic hybridization, etc., we can overcome the fertilization barriers encountered during distant hybridization (**Figure 2**).

### Gene pools of legume crops

Chickpea (*Cicer arietinum* L.) is the only domesticated species of the genus *Cicer* and was evolved from its immediate wild progenitor, *C. reticulatum* Ladiz. through natural selection. The genus *Cicer* consists of 46 species, of which 10 are annual and 36 are perennial in nature (Harlan and de Wet, 1971; Smýkal and Konečná, 2014; Toker et al., 2021). Of the total 10 annual species, nine were identified initially. However, one annual species, *C. turcicum* Toker, Berger & Gokturk, was introduced by Toker and co-author in 2021 while working with germplasm collection from east and south-east Anatolia (Toker et al., 2021). Among the 46 species, *C. arietinum* and *C. reticulatum* were classified as GP-1, *C. echinospermum* P.H. Davis and *C. turcicum* as GP-2, and remaining all species as GP-3 (**Supplementary Table 1**). Furthermore, group of scientists from ICRISAT, Hyderabad (International Crops Research Institute for the Semi-Arid Tropics), used EST (expressed sequence tag) libraries to classify several *Cicer* species into GP-1 (viz., *C. arietinum*, *C. echinospermum* and *C. reticulatum*), GP-2 (viz., *C. pinnatifidum* Jaub. & sp., *C bijugum* K.H. Rech. and *C. judaicum* Boiss), and GP-3 (viz., *C. yamashitae* Kitamura, *C. chrossanicum* (Bge.) M. Pop. and *C. cuneatum* Hochst. Ex Rich), respectively (Buhariwalla et al., 2005).

Pigeonpea (*Cajanus cajan* (L.) Millspaugh) is an important grain legume crop which includes nearly 13,200 cultivated and 555 wild accessions in the gene bank. Based on their crossability relationship with cultivated pigeonpea, all accessions were classified into three GPs. The *C. cajana* and *C. cajanifolius* (Haines) van der Maesen belongs to GP-1, whereas 10 species each belongs to GP-2 and GP-3, respectively (**Supplementary Table 1**). GP-3 was considered as a rich reservoir of useful genes which can be used to broaden the narrow genetic base of cultivated pigeonpea (Mallikarjuna et al., 2006).

Soybean (*Glycine max* (L.) Merr.) belongs to the genus *Glycine*, which has two subgenera, i.e., *Glycine* and *Soja.* The subgenus *Glycine* has 26 perennial species native to Australia (Chung and Singh, 2008). Whereas, the subgenus *Soja* includes two cross-compatible, annul species i.e., cultivated soybean (*G. max*) and its wild progenitor (*G. soja* Sieb.) (Singh and Hymowitz, 1987). Furthermore, all the germplasms of the genus *Glycine* have been classified into GP-1 and GP-3, with no identified species belonging to GP-2. GP-1 includes all the cultivars and landraces of *G. max* and their wild progenitor, *G. soja*, which produces vigorous hybrids with 100% fertility (Palmer and Hymowitz, 2016). Majority of the *Glycine* species belong to GP-3 as they have not been hybridized with GP-1 (**Supplementary Table 1**). However, a methodology for producing fertile crosses has been devised to introgress useful genes from GP-3 (i.e., *G. tomentella* Hayata; 2n = 78) to GP-1 (*G. max*) (Singh and Hymowitz, 1987).

Groundnut (*Arachis hypogaea* L.) belongs to the genus Arachis, with 80 species. All groundnut species are divided into total nine taxonomic sections on the basis of their sexual compatibilities, morphological and cytogenetic features, and geographic distributions (Krapovickas and Gregory, 1994). The section *Arachis* contains, *A. hypogaea* and *A. monticola* Krapov. & Rigoni, as GP-1, most closely related wild species as GP-2, and wild species belonging to other sections (viz., *Procumbentes* and *Rhizomatosae*) as GP-3 (**Supplementary Table 1**).

Wild species/sub-species of the genus *Lens* act as the potential sources of genetic diversity in cultivated lentil (*Lens culinaris* Medik.). The genus *Lens* has seven closely related taxa and were categorized into four GPs (**Supplementary Table 1**) (Wong et al., 2015). It has been reported that, viable hybrids were obtained by crossing sub-species *culinaris* with *orientalis* and *odomensis* (Gupta and Sharma, 2006). Furthermore, it has been shown that *L. orientalis* (Boiss.) Hand.-Mazz and *L. odemensis* (Ladiz.) are crossable with cultivated lentils and may share the common gene pool (Ladizinsky and Muehlbauer, 1993).

Common bean (*Phaseolus vulgaris* L.) is the important legume crop with limited germplasm characterization. Over the course of its evolution and domestication, the common bean eventually formed two separate GPs, namely the Mesoamerican and the Andean (Smartt and Simmonds, 1995). Furthermore, on the basis of phaseolin seed protein, allozymes, nucleotide sequences, and molecular markers, different germplasms of common beans were assigned to the Mesoamerican and Andean GPs. In addition, while studying the relationships between different *Phaseolus* species, Debouck has classified all the wild relatives of common bean into GP-1, GP-2, and GP-3 (Debouck, 1999).

The faba bean (*Vicia faba* L.) is an important legume crop. On the basis of seed weight, seed shape, and pod characteristics, faba bean has been divided into four groups, i.e., major, equine, minor, and paucijuga (Cubero, 1974). The genus *Vicia*, with approximately 200 species, has much genetic variation. Most of these variations were not explored in the recent past due to the presence of several incompatibility barriers (Hanelt and Mettin, 1989).

Pea (*Pisum sativum*) belongs to the genus, *Pisum*, present in the tribe *Fabeae*, and is the oldest domesticated crop, with an estimated domestication of about 10,000 years ago (Smýkal et al., 2011). Selection followed by domestication resulted in the accumulation of large number of pea accessions (more than 10,000 accessions) in the genebank (Kreplak et al., 2019). Diversity analysis among the germplasm collections using bayesian analysis of population structure classified the cultivated peas into Afghanistan, Ethiopia and China group. These results indicate the presence of large genetic variation in the cultivated gene pool. In addition, diversity analysis using retrotransposons separated the wild species and subspecies such as, *P. fulvum* Sibth and Smith, *P. sativum subsp. elatius* (M.Bieb.) Asch. & Graebn., and *P. abyssinicum* A. Braun from their cultivated genepools (Smýkal et al., 2011). Pea includes all the cultivated forms and one of its wild relatives (viz., *P. elatius*) in the GP-1 (Hanelt and Mettin, 1989). Field pea produces fertile hybrids with great difficulty when crossed with *P. fulvum* and *P. abyssinicum*, the members of GP-2 (Smýkal et al., 2011; Weeden, 2018; Kreplak et al., 2019; Sari et al., 2021). However, it doesn’t produce any viable offspring upon hybridization with *Vavilovia formosa* (Stev.), a member of GP-3 (Golubev, 1990). This is owing to the presence of strong reproductive barriers between these species.

The genus *Vigna* comprises of five sub-genera with more than 100 wild species (Schrire et al., 2005). At least ten agriculturally important crops have been domesticated from three (viz., *Vigna*, *Plectrotropis*, and *Ceratotropis*) of these five sub-genera in Asia, Africa, and America. Domesticated cowpea (*Vigna unguiculata* (L.) Walp.) belongs to the section *Catiang* of sub-genus *Vigna* (Takahashi et al., 2016), whereas its wild relatives belong to the sections *Macrodontae* and *Reticulatae*. Two cowpea groups, *sesquipedalis* (yard-long bean) and *unguiculata* (grain and vegetable cowpea) differ for their pod length and are cultivated in Asia and central Africa, respectively (Garcia-Oliveira et al., 2020). The subgenus *Ceratotropis* of the genus *Vigna* is the most important taxonomic group from which seven agriculturally important crops have been domesticated (Takahashi et al., 2016). They included moth bean, minni payaru, green gram, black gram, creole bean, rice bean, and adzuki bean. Green gram (*V. radiate* (L.) R. Wilczek) and black gram (*V. mungo* (L.) Hepper) are the two most important legumes, which were believed to be originated from the single ancestor (Das et al., 2018). Later, it has been proved that, green gram and black gram has the independent origin from *V. radiata* var. *sublobata* (Roxb.) Verdc and *V. mungo* var. *silvestris* Lukoki, Marechal & Otoul, respectively (Kumar et al., 2011). Phylogenetic analysis revealed that, *V. indica* T.M.Dixit, K.V.Bhat & S.R.Yadav closely related to moth bean (*V. aconitifolia* (Jacq.) Marechal) (Takahashi et al., 2016), *V. tenuicaulis* N.Tomooka & Maxted, *V. minima* (Roxb.) Ohwi & H.Ohashi and *V. stipulacea* (Lam.) Kuntze with rice bean (*V. umbellata* (Thunb.) Ohwi & H.Ohashi) (Vir et al., 2010), and *V. umbellata* with section *Angulares* species such as, *V. exilis* Tateishi & Maxted*, V. hirtella* Ridl*, V. tenuicaulis* N.Tomooka & Maxted, *V. minima*, *V. nepalensis* Tateishi & Maxted, *V. Ruikiuensis* Doi. and *V. nakashimae* (Ohwi) Ohwi & H.Ohashi (Kaga et al., 2002). List of species belonging to GP-1, GP-2, and GP-3 of cowpea, green gram and black gram are presented in **Supplementary Table 1**.

## Back to wild

Agricultural practises have domesticated hundreds of crop plants from their wild relatives with altered phenotype and genotype (Gros-Balthazard and Flowers, 2021). During the initial process of domestication, plants chosen for cultivation were indistinguishable from their wild relatives. However, over the period of artificial selection, cultivated plants diverged from their wild species due to the accumulation of domestication syndrome traits. During domestication, artificial selection for economically important traits (such as increased grain yield, self-fertility, non-shattering, etc.) reduced the crop’s genetic diversity. Furthermore, domestication greatly reduces the effective population sizes which altered the genotype frequencies in the population. It also caused domestication bottleneck in the population, which further reduced the genetic diversity in the crop plants (Gros-Balthazard and Flowers, 2021). Reduced genetic diversity coupled with changing climatic conditions and surging pests has become a curse to agricultural production. Therefore, it became essential for plant breeders to dig out the useful genetic resources stored in the crop wild relatives (CWRs) to enhance genetic gain and achieve targeted food requirements.

Favourable alleles stored in the wild repositories can be explored to break the yield plateau (**Figure 2**). It has been evident from several crops that the wild relatives serve as the potential sources of economic traits (including yield and tolerance to biotic and abiotic stresses) (Dempewolf et al., 2017). The potential donors of wild species can also be utilised in legume breeding programmes to develop climate-resilient, high-yielding legumes. Some of the examples of introgressing beneficial genes from wild species into legumes have been presented in **Supplementary Table 2**.

Huge germplasm collections of Chickpea (~14,803), Groundnut (~13,831), Pigeonpea (~11,797), Soybean (~5,489), Pea (~4,583), Green gram (~4,325), Common bean (~4,149), Cowpea (~3,928), Horse gram (~3,133), and Rice bean (~2,210) are stored in the National Bureau of Plant Genetic Resources (NBPGR), New Delhi (http://www.nbpgr.ernet.in/Research_Projects/Base_Collection_in_NGB.aspx). A huge amount of chickpea germplasm (including landraces, genetic stocks, wild *Cicer* species, modern cultivars, and mutants) is conserved at various gene banks (https://www.croptrust.org/). Globally, ICRISAT, Hyderabad has the largest collections of cultivated chickpeas (19,959 accessions) and wild *Cicer* species (308 accessions from 18 species) from 60 countries. These reservoirs can be utilised in breeding programmes to boost the genetic gain in legumes. However, lack of appropriate germplasm characterization and pre-bred lines may limit their effective utilization in breeding programmes. Therefore, it has become indispensable to characterize the crop germplasm and develop the pre-breeding population.

## Trait introgression and pre-breeding

New combinations of genes resulting from spontaneous hybridization (between cultivars and their wild relatives) and trait introgression are considered as the major avenues for gene flow to expand the genetic diversity of cultivated crops (Anderson, 1961; Arnold, 1992; Ellstrand et al., 1999). However, identifying the extent and significance of such natural introgression is uncertain (Jarvis and Hodgkin, 1999). Therefore, the deliberate introgression of desirable traits into cultivars has become an integral part of plant breeding since 1949, when Dr. Edgar Anderson proposed conventional breeding practises such as backcrossing for trait introgression (Anderson, 1949). Distant hybridization in combination with pre-breeding is considered a handy tool for introducing specific traits from wild species into elite lines to broaden their genetic base (Simmonds, 1993; Gill et al., 2011).

**Figure 3:**
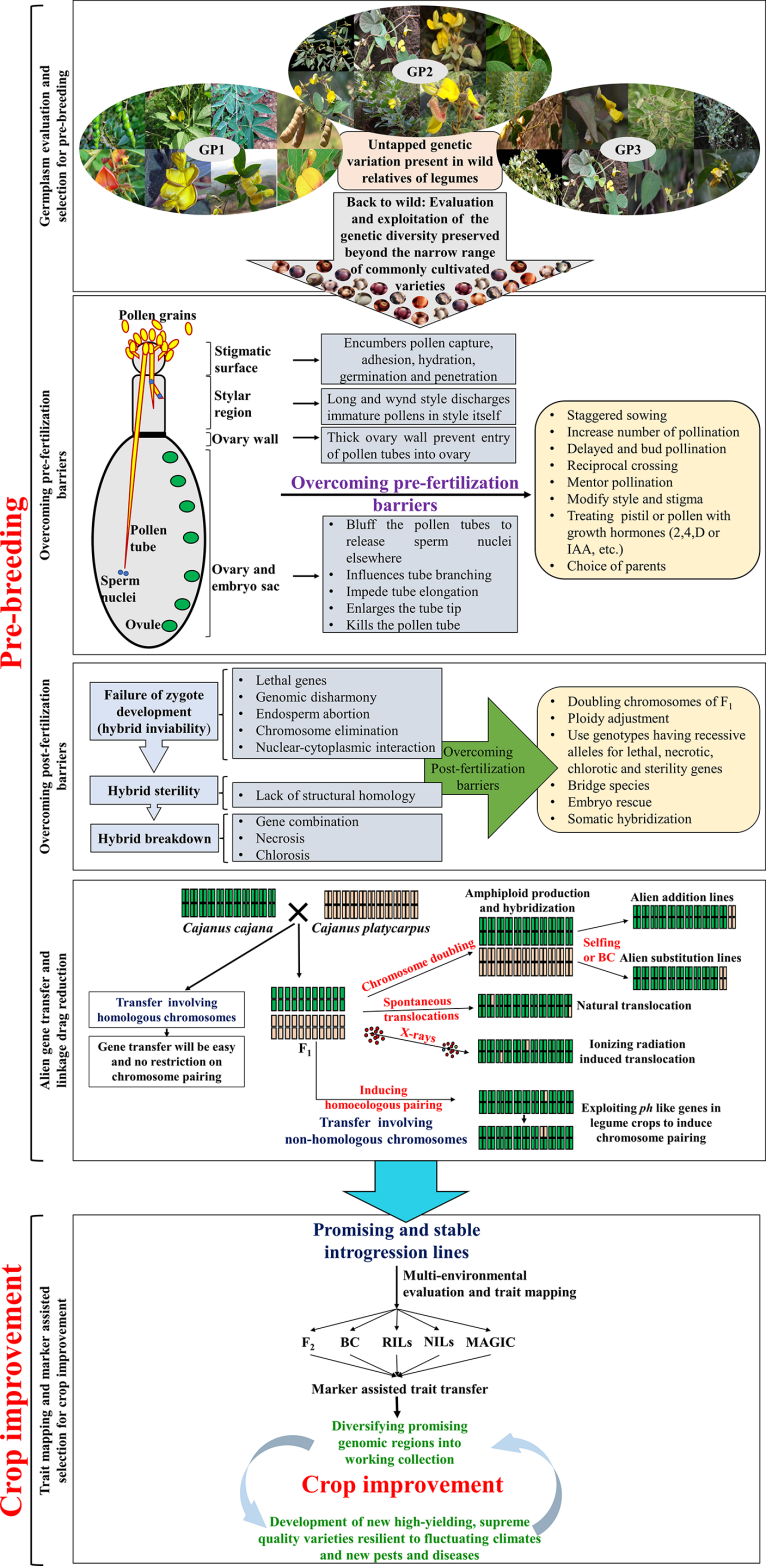
Strategies for pre-breeding and crop improvement in legumes.

Pre-breeding involves all the activities associated with identification of desirable traits/genes from unadapted germplasm (i.e., exotic/wild donor parents that cannot be used directly in breeding programmes) and transfer of these traits/genes into well-adapted, cultivar backgrounds (i.e., recipients). Pre-breeding offers a great opportunity to broaden the GP-1 by utilising genetic variability available in the wild species. This will ensure a steady flow of new and useful genetic variability into the breeding pipelines for the development of new cultivars with high levels of resistance and wider adaptability (Shimelis and Laing, 2012). Pre-breeding activities must be initiated to develop stable ILs by transfering desirable genes and quantitative trait loci (QTLs) present in the wild relatives and landraces (Jarvis and Hodgkin, 1999). These ILs can be directly crossed with working collections (**Figure 3**) to develop high-yielding, supreme quality varieties, with high tolerance to fluctuating climates and new pests and diseases (Sharma, 2017).

As mentioned earlier, huge genetic variation is available in the wild relatives of legumes with several important traits. Despite their potential applications in crop improvement, legume breeders are reluctant to use wild species in their breeding programmes. This is owing to a lack of proper germplasm characterization, cross-incompatibility, and associated linkage drags (**Figure 3**). Linkage drag is the reduction in fitness of an individual due to the introduction of deleterious genes along with desirable genes during alien introgression. Linkage drag may introduce undesirable traits such as delayed maturity, pod shattering, unappealing seed coat colour and texture, anti-nutritional factors, etc., along with the trait of interest. Several backcrosses are required to reduce such linkage drags, which is the most time and resource-consuming part of IL development process (**Figure 3**) (Sharma et al., 2013; Gudi et al., 2020). However, breeders can employ modern breeding approaches, including marker-assisted backcrossing (MABC), to purge the linkage drag and also to speed up the recovery of recurrent parent genome (**Figure 2**).

## Role of pre-breeding and alien gene introgression in legume crops

### Chickpea

Chickpea is a cool-season, low-input demanding crop that ranks third in world production among the legumes (Varshney et al., 2021). Chickpeas are cultivated in more than 50 countries on residual soil moisture. India, being the world’s largest producer and consumer of chickpeas, will produce approximately 12 million tonnes annually (Varshney et al., 2021). According to the World Health Organization, chickpeas are promoted by nutritionists and food manufacturers as plant-based healthy foods. This increased the worldwide demand for chickpea consumption. However, the low-yielding ability of chickpea cultivars, fluctuating climates, and surging pests and diseases prevented them from meeting the world demand. In this regard, there is a need to improve the productivity of chickpeas to meet the market demand. Several efforts were made to improve the productivity of chickpeas by breeders in the past by developing several high-yielding varieties.

Despite these efforts, global chickpea productivity has not increased significantly, which is owing to the narrow genetic base of the GP-1 (Mallikarjuna et al., 2007; Varshney et al., 2012b). Alien introgression may give the opportunity to broaden the genetic base by exploiting germplasm resources present in the wild *Cicer* taxa. Furthermore, multi-parent populations such as MAGIC (Multiparent advanced generation inter-cross) and NAM (Nested Association Mapping) populations may enhance the allelic diversity and may also help in the inclusion of novel recombinants in GP-1 (Varshney et al., 2019a). In addition, integrating advanced genomic tools with conventional breeding may bring a paradigm shift in the chickpea introgression programmes (**Figure 2**). The availability of genomic resources such as draft-genome assemblies, sequence-based molecular markers, ultra-high-throughput genotyping platforms (re-sequencing), quality check panels, and translational genomics has bridged the genotype-phenotype gap and enhanced gene introgression (Rasheed et al., 2017). Genomics-assisted breeding, haplotype-based breeding, and gene editing may further enhance trait introgression and may also help in purging the deleterious alleles coming from the wild species (Varshney et al., 2021).

Successful deployment of alien gene introgression has been reported in chickpea for developing improved varieties (Bharadwaj et al., 2021), pre-breeding lines (Malhotra et al., 2002), genetic stocks (Singh et al., 2021b), and mapping populations (Singh et al., 2021b; Lakmes et al., 2022). Several attempts were made to introgress the desirable traits from wild accession of chickpea into the cultivar background. Some of them includes: (i) Agronomic traits, such as early flowering (Lakmes et al., 2022), early maturity (Robertson et al., 1997), seed number per plant (Gupta et al., 2017), and seed yield (Gupta et al., 2017); (ii) High grain protein (Singh and Pundir, 1991); (iii) Biotic stresses, such as bruchids (Toker et al., 2021), cyst nematode (Ahmad et al., 2013), pod borer (Sharma et al., 2006), root knot and lesion nematode (Reen et al., 2019), leaf minor (Chrigui et al., 2020), *Fusarium* wilt (Ahmad et al., 2013), botrytis (Kaur et al., 2013), and *Ascochyta* blight (Li et al., 2017); (iv) Abiotic stresses, such as salinity (Srivastava et al., 2016), cold (Mir et al., 2021), heat (Toker et al., 2021), and drought (Barmukh et al., 2022). These ILs have boosted chickpea productivity by harmonising crop phenology and improving tolerance to multiple stresses (Malhotra et al., 2002; Singh et al., 2005; Barmukh et al., 2022). Among the available C*icer* species, *C. reticulatum* is the only species that readily crossable with *C. arietinum*. However, remaining species need specialised techniques such as embryo rescue to produce viable hybrids (Mallikarjuna and Jadhav, 2008). The “ILWC119” accession of *C. reticulatum* has been successfully used in developing cyst nematode-resistant lines, ILC10765 and ILC10766 (Malhotra et al., 2002). In addition, *C. reticulatum* was used to develop high-yielding (about 6-17% higher seed yield over best check varieties) Desi and Kabuli ILs with enhanced resistance to wilt, foot rot, and root rot (Singh et al., 2005). Successful interspecific crosses between *C. arietinum* and *C. reticulatum* (Ladizinsky and Adler, 1976), encourage chickpea breeders to make crosses between *C. arietinum* and *C. echinospermum* (Pundir and Mengesha, 1995). These two species (viz., *C. reticulatum* and *C. echinospermum*) were used to increase variability, transfer genes for several abiotic (viz., cold tolerance) and biotic (viz., wilt, root rot, ascochyta blight, and botrytis grey mold) stresses, and also to exploit higher heterosis (Koseoglu et al., 2017; Singh et al., 2018b) (Sari et al., 2022; Singh et al., 2022b).These two species were used to develop promising early maturing ILs with improved agronomic traits, including seed yield (Sari et al., 2022; Singh et al., 2022b). Furthermore, interspecific hybridization between commercial variety of the cultivated chickpea (viz., Gokce), and wild accessions belonging to *C. reticulatum* and *C. echinospermum* species helps to identify the QTLs associated with flower initiation and flower colour in NAM population (Lakmes et al., 2022). In addition to *C. reticulatum* and *C. echinospermum*, several successful interspecific crosses were made between chickpea and *C. cuneatum* Hochst. ex A. Rich. (Singh and Singh, 1989), *C. judaicum* Boiss. (Singh et al., 1999), *C. pinnatifidum* Jaub. & Spach (Badami et al., 1997), and *C. bijugum* Rech.f. (Mallikarjuna and Jadhav, 2008). Furthermore, through distant hybridization, the Punjab Agricultural University, Ludhiana have developed a high-yielding chickpea variety (PBG-8) with moderate resistance to *Botrytis* grey mould and wilt (Anonymous, 2022).

MABC has been employed to develop drought tolerant chickpea varieties (Pusa-362, Pusa-372, and DCP 92-3) (Bharadwaj et al., 2021) by transferring “*QTL-hotspots*” harbouring QTLs for several root traits from ICC-4958 (*C. arietinum*) into JG-11 (Varshney et al., 2013a). This approach has also succeeded in introgressing two genes (*foc2* and *foc4*) for *Fusarium* wilt resistance and pyramiding three genes, i.e., one for *Ascochyta* blight and two for *Fusarium* wilt resistance (*foc1* and *foc3*), respectively (Varshney et al., 2013b). *QTL-hotspot* harbouring the adaptive alleles for multiple traits under drought stress conditions were introgressed from ICC-4958 into five elite chickpea cultivars and were validated by developing SNP based KASP markers (Barmukh et al., 2022). Furthermore, MAS helped to introgress leaf miner resistance genes from *C. reticulatum* to chickpea (Chrigui et al., 2021). The Translational Chickpea Genomics Consortium (TCGC) was set up during 2016 for major chickpea growing states in India with the goal of deploying modern genetic approaches to breed improved varieties. TCGC developed ILs with enhanced drought tolerance and *Fusarium* wilt resistance in the genetic background of ten elite cultivars (Palakurthi et al., 2021). A large number of chickpea germplasm collections are stored at NBPGR, New Delhi, and ICRISAT, Hyderabad. Despite the presence of huge chickpea accession repositories, their utilisation in breeding programmes remains limited due to paucity of information regarding their nature of diversity, economic use, and preferential exploitation of GP-1 due to the presence of cryptic genetic variation and linkage drag with other GPs.

### Pigeonpea

Pigeonpea is an important grain legume crop in the semi-arid tropics and is grown in about 50 countries. It is the first non-industrial food legume crop for which a draft genome sequence has been developed (Varshney et al., 2012a). In pigeonpea, all parts of the plants have economic importance. For instance, seeds are consumed as green vegetables and *dhal*, seed husks used as cattle feed, dry stems used as fuel-wood, raw material to make huts and baskets, and soil mulch, and green leaves used for green manuring and medicinal purpose (traditional) (Halladakeri et al., 2022). Seeds are rich source of proteins and essential amino acids such as methionine, lysine, and tryptophan (Saxena et al., 2010). Pigeonpea has a tap root system, which helps it grow under resource-limited marginal soils (Varshney et al., 2012b).

The poor yielding potential of the pigeonpea genotype is owing to its lower harvest index coupled with limited selections for superior types (Varshney et al., 2010). The narrow genetic variation present in the pigeonpea can be widened by introducing genomic regions from the wild, un-adapted germplasm into the cultivars (Hoisington et al., 1999). Pre-breeding provides an excellent opportunity to introgress desirable genes from GP-2 and GP-3 into GP-1 (**Figure 2**). *Cajanus* species are reservoirs of untouched diversity that can be explored to enhance the disease and insect pest resistance (Singh et al., 2020a), abiotic stress tolerance (drought, salinity, and temperature) (Srivastava et al., 2006), and production potential of cultivars (Dwivedi et al., 2008). For instance, resistance to pod borer has been successfully introgressed from GP-2 and GP-3 in pigeonpea (Singh et al., 2020a). These ILs exhibit variability for several agronomic traits and were also shown resistance to phytophthora blight, bruchid, and pod fly (Mallikarjuna et al., 2011; Jadhav et al., 2012). The *C. scarabaeoides* (L.) Thouars, a wild relative of pigeonpea has agronomically superior characteristics, including dwarfness, the number of fruiting branches and pods per plant, and resistance to pod borers and phytophthora stem blight (Upadhyaya et al., 2013; Singh et al., 2020a). The yield-attributing genes from *C. scarabaeoides* have been successfully introgressed into the Pigeonpea (Singh et al., 2018a; Sharma et al., 2019; Singh et al. 2020a; Singh et al. 2021a). The wild accession of pigeonpea also served as the important source of male sterility genes. Some of them includes, *C. sericeus* (Benth. ex Baker) Maesen for A1 CMS, *C. scarabaeoides* for A2 CMS, *C. volubilis* (Blanco) Blanco for A3 CMS, *C. cajanifolius* (Haines) Maesen for A4 CMS, *C. cajan* for A5 CMS, *C. lineatus* (Wight & Arn.) Maesen for A6 CMS, *C. platycarpus* (Benth.) Maesen for A7 CMS, *C. reticulatus* (Aiton) F.Muell for A8 CMS, and *C. lanceolatus* (W.Fitzg.) Maesen for A9 CMS (Sharma et al., 2019). Pigeonpea’s photoperiod sensitive nature (short-day) limited its cultivation to specific locations and seasons. In this direction, photoperiod-insensitive and extra-early flowering ILs were developed by crossing pigeonpea with its wild relatives, C. platycarpus, C. volubilis, C. acutifolius (F.Muell. ex Benth.) Maesen, and C. cajanifolius (Hussain et al., 2022). These ILs could be exploited in photoperiod-insensitivity breeding programmes to develop cultivars for new environments. Furthermore, Surekha and co-authors developed salt-tolerant transgenic pigeonpea lines (with improved proline accumulation) by transferring P5CSF129A gene present in V. aconitifolia (Surekha et al., 2014). The Punjab Agricultural University, Ludhiana have developed a *C. scarabaeoides* derived introgression line, AL-1747, having moderate level of tolerance to pod borer and registered as a genetic stock by NBPGR, New Delhi (Dhillon et al., 2020).

### Soybean

Soybeans have been considered as the most important crop, with a wide range of applications. It originated in China, with about 23,000 cultivars grown in Asia, the USA, Brazil, India, and other countries (López-López et al., 2010). It is primarily grown for its meal, but it is also a rich source of edible oil (Sugiyama et al., 2015). World trade counts soybeans as the top oilseed crop, with an annual production of nearly 35 million tonnes (**Figure 1**). However, fluctuating climates, unpredictable rainfall, and emerging pests and diseases pose a serious threat to the quality and productivity of soybeans.

Genetic diversity in soybeans has been greatly diminished as result of domestication and artificial selection (Hyten et al., 2006). The negative correlation of oil and protein content with yield and its attributing characteristics will further limit the simultaneous improvement of quality and yield parameters in soybean. Wild *Glycine* species are potentially source of genetic diversity for soybean. There is a considerable genetic divergence among the species of the genus *Glycine*, and attempts to transfer this variability through interspecific hybridization have generally met with little success (Broué et al., 1982). *G. max* is able to hybridise with *G. soja* to produce fertile hybrids. The impediment is that *G. soja* has several undesirable genetic traits, such as lodging susceptibility, lack of complete leaf abscission, seed shattering, and small seeds. Further efforts were made to transfer genes from GP-3 using embryo rescue and other techniques.

The wild accessions of *G. soja* has been identified as a source of resistance genes for yellow mosaic virus (YMV) (Singh et al., 1974), nematodes (Bauer et al., 2007), aphids (Zhang et al., 2017), and brown spot (Lim and Hymowitz, 1987) It addition, it is also have desirable traits for abiotic stress tolerance including drought, cold, salinity, and heat (Ning et al., 2017). Other wild species which serve as a source of genes includes, *G. canescens* F.J.Herm. for powdery mildew (Mignucci and Chamberlain, 1978), *G. tomentella* Hayata for nematode and leaf rust (Riggs et al., 1998), and *G. latifolia* (Benth.) Newell & T.Hymowitz for stem rot (Hart et al., 1991). These species are evaluated for target traits and accessions with desirable genes were successfully employed in developing ILs harbouring alien genome fragment. For instance, Singh and co-authors identified two YMD free accessions (PI-171433 and *G. formosana* Hosok.) after screening 5,000 lines (Singh et al., 1974). In addition, YMD resistance genes exhibiting inhibitory gene action were identified from F_2:3_ and BC_1_F_2_ mapping populations (Khosla et al., 2021). A yield-related QTLs present in *G. soja* was identified on chromosome-14 and was found to increase the soybean yield by 9.4 per cent (Concibido et al., 2003). Genes for flowering, plant height, lodging, and yield-related traits were also introgressed successfully from *G. tomentella* (Akpertey, 2015). MABC has been employed to introgress null mutants of *Kunitz trypsin inhibitor* (*kti*) genes to develop trypsin inhibitor free lines (Maranna et al., 2016).

### Groundnut

Groundnut, also known as peanut, is major oil, food, and feed legume crop cultivated in tropical and subtropical regions of the world. The cultivated groundnut is an allotetraploid that originated from diploid progenitors, *A. duranensis* Krapov. & W.C.Greg. (AA) and *A. ipaensis* Krapov. & W.C.Greg. (BB) (Kochert et al., 1996). The kernels are rich source of oil, protein, vitamins (E, K, and B group), minerals, antioxidants, biologically active polyphenols, flavonoids, and isoflavones. The genus *Arachis* has nine sections with different genomes. The wild species of *Arachis* have huge genetic variations for agronomically important traits. Due to ploidy or genomic differences, this variability is not immediately available for groundnut improvement (Milla et al., 2005). Furthermore, genomic incompatibilities limit the introgression of useful genes from diploid wild species into cultivated groundnut. However, such barriers can be overcome by chromosome doubling and subsequent backcrossing.

A high level of resistance to rust was successfully transferred from *A. cardenasii* Krapov. & W.C.Greg. to cultivated peanuts (Vindhiya Varman, 1999). Leaf spot resistance genes and yield-attributing traits present in the wild accessions of *A. cardenasii*, *A. batizocoi Krapov. & W.C.Greg*, *A. diogoi Hoehne*, *A. magna Krapov., W.C.Greg. & C.E.Simpson*, *A. monticola*, *A. stenosperma Krapov. & W.C.Greg.*, and *A. valida* Krapov. & W.C.Greg. were identified/transferred by crossing with tetraploid *A. hypogaea* (Stalker and Wynne, 1979; Holbrook et al., 2002; Leal-Bertioli et al., 2012). Interspecific hybridization and chromosome doubling of diploid species (*A. batizocoi* Krapov. & W.C.Greg. and *A. duranensis*) with *A. hypogaea* help to develop an IL (viz., ICGV-86699) resistance to multiple diseases (Reddy et al., 1996). Genetic variations for petal colour, plant type, dense canopy, erect growth habit, and stem and leaflet hairs were created by crossing *A. hypogaea* with two wild species, *A. ipansis* and *A. duranensis*. These variations were then introgressed into cultivars to incorporate the several agronomic traits (Suassuna et al., 2020). Recently, the CRISPR/Cas9 (Clustered Regularly Interspaced Short Palindromic Repeats) system was used to explore the functions of *AhNFR1* and *AhNFR5* genes, responsible for nodulation. Among these two genes, the *AhNFR5* gene validates the function of nodule formation in peanut (Shu et al., 2020).

### Lentil

Lentil is a self-pollinating, annual, winter crop. The yield stagnation in lentil has been attributed to the use of indigenous microsperma germplasm (*Pilosae* type) and repeated use of the same genotypes in breeding programmes (Kumar et al., 2003). The narrow genetic base can be broaden by introducing exotic germplasm resources of diverse origins into the breeding programme (Kumar et al., 2014). While achieving this, the interspecific crosses were made successful either by applying growth hormones like gibberellic acids (GA3) (Ahmad et al., 1995) or by employing embryo rescue (Gupta and Sharma, 2005). For instance, application of 100 ppm GA3 immediately after pollination produced fertile interspecific hybrids. Similarly, embryo rescue was used to overcome the post-fertilisation barriers in crosses between *L. culinaris* and *L. tomentosus* Ladiz. and two pre-breeding lines, ILWL-90 and ILWL-120 were produced (Suvorova, 2014). Furthermore, the technique was used to introgress anthracnose and *Orobanche* resistance genes from *L. ervoides* (Brign.) into the cultivars (Tullu et al., 2006).

*Lens* species have emerged as a valuable source of genes for resistance to anthracnose and *Orobanche* (*L. ervoides* (Brign.) Grande) (Tullu et al., 2006), resistance to *Stemphylium blight* (*L. lamottei Czefr.* and *L. ervoides*) (Podder et al., 2013), resistance to multiple diseases (*L. nigricans* (M.Bieb.) Webb & Berthel. and *L. ervoides*) (Pratap and Kumar, 2014), architectural traits (such as phenology, plant growth habit, and biomass) (*L. ervoides*) (Tullu et al., 2013), extra early maturity and photo-insensitivity (*L. odemensis*, *L. orientalis*, and *L. ervoides*), high-micronutrient content (*L. nigricans.* ILWL15, *L. culinaris*. ILWL480, and *L. ervoides*. ILWL401) (Kumar et al., 2018), flowering time (*L*. *odemensis*) (Polanco et al., 2019), yield and yield attributing traits (*L. lamottei* and *L. orientalis*) (Gupta and Sharma, 2006), and tolerance to drought (*L. odemensis*, *L. ervoides*, and *L. nigricans*) (Hamdi and Erskine, 1996), heat (*L. culinaris* ssp. *culinaris*) (Roy et al., 2012) and salinity stresses (*L. orientalis*) (Anonymous, 2014). Distant hybridization helps to transfer genes from these wild relatives into cultivars and also helps to identify transgressive segregants for agronomically important traits (Singh et al., 2013c). Furthermore, the Punjab Agricultural University, Ludhiana has developed a high-yielding lentil variety (LL-1373) with resistance to rust and pod borer by crossing IPL-406 and FLIP-2004-7L (Singh et al., 2020b).

Soil salinity affects the nodulation and nitrogen fixation in lentils. Collaborative research of ICAR, New Delhi (Indian Council of Agricultural Research) and ICARDA, Lebanon (International Center for Agricultural Research in the Dry Areas) identified the salt-tolerant accessions of *L. orientalis* (ILWL-297, ILWL-368, ILWL-371, ILWL-417, and IG-136670) (Anonymous, 2014). Successful crosses were made between *L. orientalis* and *L. ervoides* at ICARDA to develop pre-breeding lines for resistance to diseases, phenology, micronutrients, plant habit, and other important agronomic traits. Introducing these pre-breeding lines into the hybridization programme resulted in increased yield (>40%), micronutrient content, and reduced generation time (80-100 days) (Kumar et al., 2020). Recently, the first interspecific genetic map of *L*. *culinaris* cv. Alpo and *L*. *odemensis* was constructed by using RNAseq methodology (Polanco et al., 2019). Similarly, QTLs for flowering time (on chromosome-6), seed size (on chromosomes-1 and 5), and *Ascochyta* blight resistance (on chromosome-6) were mapped in the interspecific crosses.

### Common bean

Common bean is a popular legume crop that can be eaten as green pod vegetable or dried seeds (dry bean/Rajmash). Because of its medicinal properties, it can also be used to treat diabetes, heart problems, and bladder burn (Doria et al., 2012). The genus *Phaseolus* is native to America, with 80 identified species (Dohle et al., 2019). Among them, five species namely common bean (*P. vulgaris* L.), lima bean (*P. lunatus* L.), runner bean (*P. coccineus* L.), tepary bean (*P. acutifolius* A. Gray), and year bean (*P. dumosus* Macfad.*)*, were domesticated for cultivation. Two of these species, *P. vulgaris* and *P. lunatus*, were independently domesticated at least twice in Mesoamerica and the Andes. This suggests that some domestication traits, such as determinacy, might be selected multiple times in common beans (Kwak et al., 2012). Furthermore, during the process of domestication, cultivated annual beans have lost their indeterminacy, seed dormancy, and pod dehiscence with increased seed size and changed branching pattern (erect type) (Chacon-Sanchez et al., 2021).

Distant hybridization may facilitate transferring genes for economically important traits. However, while transferring desirable genes from wild relatives to cultivated beans, introgression and pre-breeding activities must focus on avoiding the transfer of deleterious traits (such as pod shattering, seed dormancy, seed size, lateness, and photoperiod sensitivity) (Koinange et al., 1996). In spite of the large germplasm collections of common bean (>25,000 accessions), genetic sources for tolerance to several biotic (leafhoppers, bacterial blight, golden mosaic virus, and root rots) and abiotic stresses have not yet been identified in the gene pool. However, some of these desirable traits are sufficiently expressed in other *Phaseolus* species. For instance, *P. acutifolius* is known to possess relatively high levels of tolerance to most of the abiotic and biotic stresses (Thomas et al., 1983). Therefore, repeated efforts have been made to hybridise common bean with *P. acutifolius* for successful gene transfer for desirable traits (Mejía-Jiménez et al., 1994).

Interspecific hybridization between *P. vulgaris* and *P. acutifolius* was not successful without *in vitro* embryo culture (Parker and Michaels, 1986). However, Haghighi and Ascher proposed congruity backcrossing (recurrent backcrossing of an F_1_ hybrid to each parent in alternate generations) without the aid of embryo rescue to produce interspecific crosses between these two species (Haghighi and Ascher, 1988). This cause the substantial recombination and may produce lot of variation in the backcross population (Anderson et al., 1996). However, it may encounter several difficulties when backcrossing initial F_1_s to *P. acutifolius* genotypes and also causes sterility of congruity hybrids when *P. acutifolius* is used as the last male parent (Mejía-Jiménez et al., 1994).

Interspecific hybrids of *P. costaricensis* Freytag & Debouck and *P. vulgaris* were used to isolate white mold-resistant IL (viz., VRW-32) (Singh et al., 1997). In addition, AB-QTL (advanced backcross QTL mapping) was used for simultaneous identification and transfer of QTL conferring resistance to white mold from *P. coccineus* cv. PI255956 to *P. vulgaris* cv. OR91G (Pratap et al., 2021). Resistance genes for anthracnose and bean common mosaic present in the different accessions of common bean were introgressed and pyramided in the fabada line, A3308 (Ferreira et al., 2012). Recently, pod shattering gene, *PvPdh1* (*Phaseolus vulgaris Pod dehiscence-1*) located on chromosome-3, identified from the middle American domesticated beans was used to develop shattering tolerant common bean (Parker et al., 2021).

### Faba bean

Faba bean is less resource consuming, cool season crop grown on marginal soils. It is also known as broad bean, bakala, horse bean, and tick bean (Singh et al., 2013a). Middle-East is considered as the primary center of origin, while China as the secondary center (Duc, 1997; McVicar et al., 2013). Faba bean is a rich source of proteins, vitamins, and minerals that can be grown for both food and feed purposes (Jensen et al., 2010; Karkanis et al., 2018).

Over the decades, the cultivation of faba bean has been steadily declining, and in some countries, the cultivation has become negligible. This is owing to the lower productivity and higher incidence of disease and pests in this crop (Lake et al., 2019). Some sources of resistance to pests such as seed weevils were identified in the germplasm accessions and were introgressed into the faba bean (Carrillo-Perdomo et al., 2019). Furthermore, the source of genes has been identified for downy mildew in *V. faba* and *V. narbonensis* L. (Ahmed et al., 2000), seed weevil in *V. equina* (Carrillo-Perdomo et al., 2019), aphid in *V. johannis* Tamamsch. and *V. narbonensis* (Birch, 1985), drought in *V. sativa* L. (Abbasi et al., 2014), and cold in *V. montbretii* Fisch. & C.A.Mey. (Inci and Toker, 2011). Even though several sources of resistance/tolerance genes have been identified, alien gene introgression in *V. faba* is encumbered by several incompatibility barriers. However, with the advent of molecular markers and embryo rescue techniques, the possibility of introgressing alien genes in *V. faba* has been increased (Ramsay and Kumar, 1990; Torres et al., 2010; Carrillo-Perdomo et al., 2019).

### Lupin

Lupin seeds have been used for thousands of years as food and feed. It has originated from the Old World (Mediterranean region), as well as from the New World (North America and Andean highlands) (Drummond, 2008). Lupin is investigated as potential alternative to soybean meal as a source of animal feed. They offer the possibility of reducing the quantities of imported soybean in Europe (Abraham et al., 2019). Only a few lupin species, including *Lupinus angustifolius* L. (blue lupin), *L. albus* L. (white lupin), *L. luteus* L. (yellow lupin), and *L. mutabilis* Sweet (Andean lupin) have been domesticated and are under cultivation (Gulisano et al., 2019). There are around 800 lupin species that are geographically distributed and found throughout North and South America (Kamphuis et al., 2021).

Crossing wild species with cultivated lupins is rarely successful (Wolko et al., 2011). With huge genetic variations present in lupin germplasm, Professor Clive Francis has collected some interesting lupin genotypes for resistance to anthracnose (*L. albus*), thin seed coats and pod walls (*L. angustifolius*) (Clements et al., 2005), reduced pod splitting (*L. luteus*), and high pod set (*L. albus*). Genes for several agriculturally important traits, such as, vernalization independence (*Ku* and *Julius*), low-alkaloid content (*iucundus*), reduced pod shattering (*tardus* and *lentus*), soft seededness (*mollis*), white flower colour (*leucospermus*), and anthracnose resistance (*Lanr1*), have been identified and transferred into *L. angustifolius* (Berger et al., 2013).

The quinolizidine alkaloid present in lupin seeds gave them bitter taste, which hampered their cultivation (Wolko et al., 2011). Several efforts were made to develop alkaloid-free lupins in Europe and other countries. The collection of simply inherited natural and spontaneous mutants that diminish the alkaloid content and produce alkaloid-free lupins has been the result of such intensive studies (Gladstones, 1970). Furthermore, introducing sweetness genes may reduce the alkaloid content in the seeds. However, the negative pleiotropic effects of the low-alkaloid genes, *iucundus* and *tardus*, prevented the development of shattering resistant, alkaloid-free lupin lines (Berger et al., 2013). Furthermore, there has not yet been a reported example of effective gene transfer for quantitative character in the lupin. Bringing novel approaches, such as genome-assisted or haplotype-based breeding may help to accelerate trait introgression and cultivar development in lupin (Kamphuis et al., 2021).

### Pea

Pea seeds are the rich source of protein and fiber, but have a lower level of cholesterol and antioxidants. It can be used as a vegetable (green pods), dry pea, or green manure crop and will also help in improving soil fertility by fixing atmospheric nitrogen. Peas are neglected by many farmers and will be grown on marginal lands, despite their importance in health benefits and sustainable crop production. Furthermore, due to limited genetic base coupled with changing climate conditions, wide range of biotic and abiotic stresses affects peas production. Novel genetic variations can be either exploited from the available wild relatives or can be created through mutagenesis (Tanksley and McCouch, 1997). Distant hybridization gives an opportunity to transfer useful genes present in the wild relatives and also helps in developing pre-breeding lines. Efforts were made to transfer the useful genes from the *P. fulvum* for pea weevil and Ascochyta blight (Byrne et al., 2008; Clement et al., 2009; Aryamanesh et al., 2012; Jha et al., 2016), *P. sativum ssp. elatius* for Fusarium wilt (Hance et al., 2004), and *Pisum spp* for *Mycosphaerella pinodes* (Fondevilla et al., 2005). Phenotypic evaluation of *P. fulvum* accessions identified 26 genotypes with high and intermediate resistance to pea weevil (Clements et al., 2002). Genetic analysis for seed resistance to pea weevil in the interspecific F_2_/F_3_ progenies of *P. sativum* and *P. fulvum* revealed the involvement of three genes with recessive inheritance (Byrne et al., 2008). The resistance genes were named as, *pwr1*, *pwr2*, and *pwr3*. Interspecific crosses were made between wild accession of *P. fulvum* (PI-595946 and PI-343955) and pea cultivar, Alaska-81 to transfer the resistance genes for pea weevil into the cultivar background (Clement et al., 2009). In addition, resistance QTLs for *Aschochyta* blight were identified and transferred into pea cultivar, Alfetta through intespecific hybridization with *P. fulvum* (Jha et al., 2016). However, the wild relatives of pea contain antinutritional factors such as protease and trypsin inhibitors, which interfere with the availability of bio molecules. Clemente and co-authors identified double null mutants for protease inhibitors (*TI1* and *TI2*) from wild germplasm accessions. Introgressing these mutants into cultivar background may reduce the antinutritional factors. The *P. elatius* mutant has very low seed protease inhibitory activity, and this mutation has been successfully introgressed into cultivated pea (Clemente et al., 2015). Furthermore, tapping into the germplasm of wild relatives of peas may help to produce high-yielding, climate-resilient cultivars. For instance, germplasm accessions were screened to identify major QTLs for cold and frost tolerance in pea (Lejeune-Henaut et al., 2008).

### Cowpea

*Vigna* is an important genus in the Leguminaeceae family and it helps in restoring soil fertility by fixing atmospheric nitrogen. Cowpea (*Vigna unguiculata* (L.) Walp.) is a warm-season legume crop and is a rich source of nutrients and minerals. It can be used as a pot herb or as livestock feed. It has the ability to thrive well in drought-prone areas and in poor soils. Cowpea is a self-pollinated crop with a monophyletic origin. Several insect-pests, including pod borers, pod sucking bugs, flower bud thrips, and cowpea aphids, will greatly affect the cowpea yield (Boukar et al., 2019). Wild species belong to the section *catiang*, harbours the resistance genes to pod borer, striga, aphid, etc. For instance, genetic sources for pod bug, bacterial blight, thrips, pod borer and striga are identified in *V. denkindtiana* (Monawana) and *V. sesquipedalis* (L.) Fruwirth (Koona et al., 2002; Dinesh et al., 2016a). In addition, *V. sesquipedalis* also act as source of genes for several abiotic stresses including heat and salinity (Harouna et al., 2020). Distant hybridization has the potential to introduce valuable resistance genes present in these wild species into cultivars. However, the fertilization barriers hinder their introgression (Fatokun, 2002). Several omics tools, including QTL mapping, GWAS (genome-wide association study), MAS, and MABC, can facilitate the identification of resistance sources and their introgression into cowpea (Dinesh et al., 2016b; Goufo et al., 2017). For instance, QTLs governing aphid resistance were mapped in recombinant inbred lines (RILs) produced from cowpea and its wild accession, TVuNu-1158 (Lo et al., 2018). Later, it was revealed that the aphid resistance is associated with lack of polyphenols and sucrose (Togola et al., 2020). Furthermore, in a year, while evaluating wild accessions of cowpea (viz.), Boukar and co-authors identified *V. oblongifolia* A.Rich. and *V. vexillata* (L.) A.Rich. as the resistance source to aphids, flower bud thrips, pod borers, and bruchids (Boukar et al., 2020). However, their introgression in cultivated cowpeas is limited by the presence of several incompatibility barriers. Most recently, Ji and co-authors have identified the symbiotic nitrogen fixing gene, *symbiosis receptor-like kinase* (SYMRK), through null mutants produced by CRISPR*/*Cas-9 technology (Ji et al., 2019). Gene specific markers developed from such genes can be used in MABC to transfer genes of interest in the cultivar background.

### Green gram and black gram

Green gram and black gram are the important short-duration legumes, and they can be intercropped with cereal crops to improve soil fertility (Mehandi et al., 2019). It is thought that the wild progenitor of black gram, i.e., *V. mungo* var. *silvestris*, was domesticated in India about 4,500 years ago (Chandel et al., 1984). Green gram is an extensively grown crop and is an excellent source of alimentary proteins, vitamin-B9, and minerals. Several biotic (bruchids, *Cercospora* leaf spot, powdery mildew, and YMV) and abiotic factors (heat, drought, water logging, and photoperiod) will cause severe yield reduction in these crops. Distant hybridization has allowed us to broaden the genetic base and offers the opportunity to deal with these problems.

Wild relatives of *Vigna*, has useful genes for vigorous and erect growth, sturdy stems, broad leaves, and long and profuse pods with biotic and abiotic stress tolerance (Tripathy et al., 2016; van Zonneveld et al., 2020). Wild accessions showing tolerance to bruchids (viz., *V. radiata* var. *sublobata*) (Tomooka et al., 1992), pod bug (*V. unguiculata* subsp. *dekindtiana (Harms) Verdc.*), nematode (*V. angularis* (Willd.) Ohwi & H.Ohashi) (Kushida et al., 2013), MYMD (mungbean yellow mosaic disease) (viz., *V. sublobata*) (Pal and Inderjit, 2000), and heat and salt stress (viz., *V. angularis*, *V. luteola* (Jacq.) Benth., *V. marina* (Burm.) Merr., and *V. vexillate*) were identified (Yoshida et al., 2020). Subsequently, bruchid and MYMD resistance genes present in *V. sublobata* accessions were successfully introgressed using high-throughput genotyping and phenotyping tools (Miyagi et al., 2004; Schafleitner et al., 2016). Furthermore, efforts were made to introgress genes for resistance and yield components from rice beans into green gram and black gram (Basavaraja et al., 2019). Furthermore, through distant hybridization between black gram and rice bean, the Punjab Agricultural University, Ludhiana has developed a superior yielding black gram variety (Mash-114) with resistance to MYMV, Cercospora leaf spot, and bacterial leaf spot (Singh et al., 2013b).

### Moth bean

Moth bean (*V. aconitifolia*) is largely cultivated as multi-purpose crop in arid regions of India. The prostrate, vining, and semi-trail growing patterns of moth bean help in reducing soil erosion. It can be used as a green manure crop and also helps to improve soil fertility by fixing atmospheric nitrogen. Moth bean has been considered as a vital source of amino acids, vitamins, and minerals (Kumar, 2002). Due to changing climates and the substitution of moth bean with more remunerative and secure crops, moth bean acreage and production are declining gradually. Cultivation in marginal soils with improper pest and disease management and non-availability of high-yielding cultivars further contributes to the lower productivity of moth bean (Tomooka et al., 2000; Kumar, 2002). Through interspecific hybridization, desirable genes present in the wild relatives can be introgressed into cultivated moth bean. Molecular investigations of wild relatives identified major (*qVacBrc2.1*) and minor (*qVacBrc5.1*) QTLs for seed resistance to *Callosobruchus chinensis*. Resistance genes for *C. chinensis* were further successfully introgressed into cultivars (Somta et al., 2018).

### Rice bean

Rice bean (*V. umbellata*) is an underutilized legume that has gained attention due to its numerous applications, including profitability, diversity, and agricultural sustainability. It is also known as “climbing mountain bean,” “red bean,” or “Oriental bean.” It is used as a green manure crop as well as a cover crop. Rice bean is mostly grown for beans, although they serve as vegetable (green pod), fodder, and folk medicine. Despite these benefits, rice bean is grown in fewer areas owing to lack of ideal plant types, non-synchronous maturity, seed shattering, and insufficient marketing facilities (Pattanayak et al., 2019). Furthermore, difficulty in splitting, unpleasant odour after cooking, and high flatulence makes it unsuitable for commercial cultivation.

Wild species of rice bean are rich source of genes for yield contributing traits such as pod length, pod number, and seed number (Singh et al., 2013b), low level of trypsin inhibitor (*V. tenuicaulis*) (Konarev et al., 2002), bruchid resistance, and photo- and thermo-insensitivity (IC251442) (Pratap et al., 2014). Interspecific hybridization provided the opportunity to introgress these genes into rice bean cultivars. For instance, distant hybridization was used to transfer MYMD resistance genes from *V. radiata* to *V. umbellata* (Singh et al., 2003). However, while transferring these genes, several pre-fertilization barriers and hybrid lethality were encountered in the crosses of rice bean with *V. radiata* and *V. mungo* (Kumar et al., 2007; Thiyagu et al., 2008). By employing embryo rescue techniques, Chen and co-authors succeeded in producing interspecific hybrids of *V. mungo* and *V. umbellata* (Chen et al., 1983). Furthermore, gamma irradiation of parental lines helped to increase pod setting in rice bean (Pandiyan et al., 2008).

### Horse gram

Horse gram (*Macrotyloma uniflorum* (Lam.) Verdc.) is an underutilised legume crop that originated in south-west India and it serves as a major source of vegetable protein for millions of rural residents in the Indian subcontinent (Kadam et al., 1985). Since the beginning of agriculture, it has been an essential legume and been used as source of fodder for cattle and horses (Fuller and Murphy, 2018). Despite the nutritional benefits, the area, production, and productivity of horse gram have been depleting consistently from last two decades. This has been partly attributed to indeterminacy, non-synchronous maturity, photo- and thermo-sensitivity, and poor harvest index coupled with lack of government policies (Henry et al., 2006; Aditya et al., 2019).

The genus *Macrotyloma* consists of around 25 species. Most of the *Macrotyloma* species are distributed in the African continent, including *M. axillare* (E.Mey.) Verdc. and *M. ciliatum* (Willd.)Verdc. However, *M. uniflorum*, is the only cultivated species distributed in the Indian subcontinent (Dikshit et al., 2014). Wild species of *Macrotyloma* are the source of genes for increased pod number and seed yield, profuse flowering, resistance to powdery mildew and YMV, tolerance to drought and cold (*M. axillare*) (Staples, 1966), increased protein and oil content (*M. sar-garhwalensis* R.D.Gaur & L.R.Dangwal) (Yadav et al., 2004), and higher biomass (*M. africanum* (Brenan ex R.Wilczek) Verdc. and *M. axillare*) (Chahota et al., 2013). Introgression of genes from *M. sar-garhwalensis* helps to improve the seed protein (38.37%) and lipid (10.85%) content in the horse gram (Yadav et al., 2004). Furthermore, the narrow genetic base present in the horse gram germplasm pool can be widened by employing distant hybridization (Aditya et al., 2019).

### Forage legumes

Forage legumes belong to the family Leguminosae, and they provide feed for livestock and also help in improving soil fertility through biological nitrogen fixation (Singh et al., 2018c). Forage legumes can be used as source of nutraceuticals and pharmaceuticals which help in deriving drugs for controlling diabetes and hypercholesterolemia (Cornara et al., 2016). The major breeding challenges for forage legumes includes: (i) resistance to biotic stresses (such as *Fusarium* spp., *Pythium* spp., root knot nematode, *Anthracnose*, collar rot, powdery mildew, *Stemphylium* leaf spot, *Alfalfa* leaf curl virus, and *Alfalfa* weevil) (Barbetti et al., 2019); (ii) forage persistence (adaptation, autumn dormancy, rhizomatous growth habit, and tolerance to low phosphorus content) (Bouton, 2012); and (iii) seed yield (Marshall et al., 2002). Forage legumes belong to the genus *Medicago*, *Trifolium*, and *Vigna*. Among these, *Medicago* and *Trifolium* are widely cultivated as forage crops.

Distant hybridization has been used as strategy to achieve genetic improvement in *Trifolium repens* (L.). For instance, reproductive traits (such as flowering and seed yield) from *T. nigrescens* Viv. (Marshall et al., 2002), tolerance to low phosphorus from *T. uniflorum* L. (Nichols et al., 2014), rhizomatous growth habit from *T. ambiguum* M.Bieb. (Lloyd et al., 2017), and drought and salt tolerance from *T. occidentale Coombe* (Hussain et al., 2016) have been successfully introgressed into *T. repens*. Introgressing rhizomatous growth habits increased the forage persistence of hybrids *via* stolon networks due to their ability to regenerate after grazing (Lloyd et al., 2017). Furthermore, embryo rescue was used to produce successful hybrids in incompatible crosses (i.e., between *T. alexandrinum* L. and *T. constantinopolitanum* Ser.) (Roy et al., 2004; Williams et al., 2019).

Lucerne (*Medicago sativa* L.) is possibly the world’s most important cultivated temperate perennial forage legume. *M. arborea* L. is another perennial forage legume, that serves as major source of genes for high level of heterosis (Irwin et al., 2010), alborea lines (yellow flowers, single-coil flat pods, large seeds, longevity, and tallness) (Irwin et al., 2016), and tolerance to biotic (anthracnose) and abiotic (drought and salt) stresses (Armour et al., 2008). Several breeding efforts were made to introgress alfalfa weevil resistance and yield potential of *M. rugosa* Desr. and *M. scutellata* (L.) Mill. into *M. sativa* (Mizukami et al., 2006). None of the conventional breeding approaches were able to produce successful hybrids between these cross-incompatible species. However, asymmetric somatic hybrids of *M. rugosa*-*M. truncatula* Gaertn. and *M. scutellate*-*M. truncatula* were successfully produced by protoplast fusion to transfer the genomic fragments from the these species (through intergenomic recombination) into *M. truncatula* (Tian et al., 2002).

Distant hybridization in forage legumes is encumbered by an imbalanced chromosome number, undesirable linkage drags, and pre- and post-fertilization barriers. Integrating conventional breeding with embryo rescue, protoplast fusion, genomic-assisted breeding, haplotype-assisted breeding, and genome editing tools may accelerate crop improvement in forage legumes. Available whole genome sequences of *Trifolium* (De Vega et al., 2015) and *Medicago* (Young et al., 2011) can be utilised in their breeding programmes. Furthermore, by utilizing genome editing tools, such as base and prime editing, one can perform targeted sequence insertion without any linkage drag (Wolabu et al., 2020).

## Integrated approaches for achieving higher genetic gain in legumes

Distant hybridization can be integrated with other breeding approaches such as genomics, genetic engineering, genome editing, haplotype breeding, speed breeding, and high-throughput phenotyping for accelerating trait introgression and achieving higher genetic gain in legumes (Pratap et al., 2022). Genetic gain is the improvement in the average genetic value within a population over the cycles of breeding (Hazel and Lush, 1942). According to the breeder’s equation, genetic gain is indirectly proportional to the duration of the breeding cycle (t) and dependent on genetic variation (σ^2^g), selection intensity (I), and trait heritability (h^2^). Genetic variation is the fundamental requirement for crop improvement. However, domestication followed by intensive selection and utilization of same parental lines has significantly reduced the genetic diversity in the legume gene pool. Narrow genetic diversity of legumes can be widened either by unlocking the hidden variation present in the germplasm resources or by creating novel alleles or haplotypes not present in the crop germplasms (Sinha et al., 2020).

The huge germplasm collections of different legume crops stored in the gene banks can be characterized to identify the functional genes, alleles, haplotypes, and gene networks. However, characterization of such a large collection is relatively challenging. Genomic tools and high-throughput phenotyping platforms may ease the task of screening customised germplasm (core and mini-core set) for specific traits of interest (Upadhyaya and Ortiz, 2001). Several next-generation sequencing (NGS) technologies (viz., Ion torrent and Illumina sequencing), high-throughput genotyping (viz., Microarray, MALDI-TOF, and Invader assay), and reduced representation sequencing methodologies were employed to decode plant genomes and identify the genomic regions associated with target traits. For instance, whole-genome re-sequencing of pigeonpea (using 292 accessions) and chickpea (using 429 accessions) provided insights into genome diversity and identified genomic regions associated with many agronomic traits (Varshney et al., 2017; 2019b). In addition, the reduced representation sequencing may provide genome-wide information for entire germplasm collections (Juliana et al., 2019). Furthermore, a slew of pan-genomics has sprung up as NGS technologies have improved, opening up new window for understanding crop evolution and adaptability (Zhao et al., 2018). Pan-genomes offer great opportunity to understand the role of genetic diversity and to catch up the lost genes in reference genomes during crop domestication. Pan-genomes provides a complete profile of haplotypes and allelic variations present in the populations. For instance, soybean and pigeonpea pan-genomes have identified the significant SNPs and haplotypes associated with important agronomic traits (Li et al., 2014; Zhao et al., 2020).

Specialized populations, including bi-parental (F_2_, RILs, BCs, DH, NILs, BILs, and immortalised F_2_), multi-parental (NAM and MAGIC), and unstructured populations (association panels), help to identify significant alleles (QTLs) or haplotypes. Multi-parent populations will combine allele richness and increase the mapping resolution by enhancing recombination frequency (Huynh et al., 2018). However, GWAS exploit historical recombination and linkage disequilibrium to find significant marker-trait associations. Furthermore, these genomic regions (QTLs identified) can be subjected to meta-QTL analysis, QTL fine mapping and cloning to identify genes of interest that are likely to influence the targeted traits (Gudi et al., 2022; Halladakeri et al., 2022; Tanin et al., 2022).

Novel molecular genetics tools such as transgenesis, mutagenesis (using physical or chemical mutagens) and genome editing can be effectively used to transfer favourable genes from unrelated species or produce novel genetic variations not present in the nature. Transgenesis has been successfully employed in broad range of legume species and it can potentially stack multiple genes for targeted traits (Somers et al., 2003). Mutations are sudden heritable changes (either spontaneous or induced) that occur due to altered genetic messages carried by the genes. TILLING and ECO-TILLING allow the identification of directed mutations present in the specific genes and spontaneous mutations present in the population, respectively (Till et al., 2006). Genome editing using RNAi and nucleases (viz., ZFNs, TALENs, and Cas9) is the targeted insertion, removal, and swapping technique to produce non-transgenic plants. CRISPR-Cas9 has emerged as an efficient genome editing tool with the highest efficiency in the 21^st^ century (de Maagd et al., 2020). CRISPR-Cas9 can be effectively used to purge out deleterious mutations and linkage drag, induce site-specific recombination, and can also be used perform whole genome editing (major and minor genes). The CRISPR-Cas9 system can be used in distant hybridization to knockout genes associated with fertilization barriers (such as crossability, lethal, necrotic, chlorotic, and sterility genes) or homoeologous recombination (*ph*-like and *REC* genes) (de Maagd et al., 2020). This can also be used for the functional validation of candidate genes associated with traits of interest. For instance, the CRISPR/Cas9 system was used to explore the candidate genes associated with symbiotic nitrogen fixation in groundnut (*AhNFR1* and *AhNFR5*) and cowpea (*SYMRK*) (Ji et al., 2019; Shu et al., 2020).

Identified or created genetic variations (alleles/haplotypes) can be transferred into breeding populations by employing genomics-assisted breeding tools (MAS, MABC, MARS, AB-QTL, and GS) (Varshney et al., 2005). The MAS facilitates the indirect selection of desirable plants in the early segregating generations and can also be used for rapid recovery of recurrent parent genomes in the MABC (Maranna et al., 2016; Bharadwaj et al., 2021). Recurrent selection is an efficient strategy for improving quantitative traits by increasing the frequency of desirable alleles and by maintaining the high genetic variability in the population (Hull, 1945). Employing molecular markers in recurrent selection (via MARS) will reduce the number of cycles of selection and also increase the efficiency of selection. Despite the presence of desirable genes for quantitative traits in wild relatives, their introgression and QTL mapping are hindered by linkage drag, pleiotropic effects, poor phenotypic performance, and epistatic interactions. However, by delaying the QTL analysis in later backcross generations, one can simultaneously do QTL mapping and varietal development by purging out the linkage drag, reducing epistatic interactions, and increasing selection efficiency (Tanksley and Nelson, 1996). Genomic selection (GS) uses whole-genome markers to predict the phenotype of breeding population using genome estimated breeding values (GEBVs) and thereby helps to improve the quantitative traits (Meuwissen, 2003). It has been considered as the most promising tool to achieve higher genetic gain in the breeding program.

High-throughput phenotyping platforms such as spectral reflectance provide an opportunity to utilize the vast amount of genomic data accumulating in databases. Furthermore, by combining phenomics and envirotyping with refined field trails and experimental designs, one can significantly improve heritability estimation and reduce spatial heterogeneity in the field, and, as a result, achieve higher genetic gain. However, decoding and using the information from such tremendous dataset generated from phenomics demands data science, particularly artificial intelligence and machine learning, to develop model-based breeding methods (Ahmar et al., 2020).

Higher genetic gain can also be achieved by reducing the breeding cycle *via* rapid generation advancement tools such as, single seed decent, shuttle breeding, doubled haploid (Srivastava and Bains, 2018), biotron breeding (Ohnishi et al., 2011), *in vitro* nursery (Gerald et al., 2013), and speed breeding (Watson et al., 2018). Speed breeding using protracted photoperiod and elevated temperature coupled with immature embryo culture and growth hormones has been successfully used to accomplish up to eight generations per year in lentil, seven generations in faba bean (Mobini et al., 2015), six generations in chickpea and pea (Watson et al., 2018), five generations in pea (Mobini and Warkentin, 2016), and pigeonpea (Saxena et al., 2019), and 3-4 generations in groundnut (O’Connor et al., 2013), instead of 2-3 generations under normal glasshouse conditions.

Integrating genomics and phenomics tools with modern breeding approaches (Singh et al., 2022a) will increase genetic variability, selection efficiency, and heritability. Genomic-assisted breeding tools facilitate multiple genes stacking and accelerate alien gene introgression. Furthermore, integrated approaches such as speed-MAS, speed-MABC, speed-MARS, speed-GS, and express edit may significantly reduce the breeding cycle time and archive higher genetic gain (**Figure 2**).

## Conclusion and future perspectives

Under changing climatic conditions and deteriorating arable lands, the cropping system has to be changed to achieve sustainability in farming. Legume crops that have an inherent capacity to fix atmospheric nitrogen can improve soil fertility while also ensuring food and nutritional security. Despite their economic importance and health benefits, the rate of legume improvement is negligible, which is owing to the narrow genetic base of legume crops. The huge genetic variation present in the legume germplasm resources (i.e., GP-2 and GP-3) offers an avenue to broadening the genetic base of these crops. Taking into account the number of wild accessions available in gene banks, a rational sampling of target donors would have to be achieved. Reduced representation sequencing and high-throughput phenotyping offer the advantage of characterizing all germplasm lines stored in gene banks (Juliana et al., 2019). Furthermore, pan-genomes of legume crops will provide the complete profile of all haplotypes and allelic variations for the trait of interest (Li et al., 2014). Once germplasm lines have been characterized, they can either be used directly in varietal development (through AB-QTL analysis) (Tanksley and Nelson, 1996) or can be used in generating a set of ILs. Several pre- and post-fertilization barriers encumbers the alien gene introgression and genetic improvement of legume crops. Advanced breeding and molecular techniques can be employed in distant hybridization to overcome incompatibility barriers and induce homoeologous recombination for the production of interspecific hybrids and pre-breeding lines (Pratap et al., 2018). Promising and stable pre-breeding ILs identified from the multi-environmental evaluation can be utilized in the genomic-assisted legume improvement programme to develop high-yielding, supreme quality cultivars resilient to fluctuating climates and surging insect-pests.

One of the major lessons learned from wild species is that the valuable alleles present in CWRs can only be identified when they are incorporated into cultivar background. Alien gene introgression have offered us dividend results in chickpeas. Still, the chickpea hybridization programme needs meticulous planning and implementation for full utilization of wild resources to break the yield plateau. Wild species of pigeonpea offer the potential use in hybridization programmes for introgression of multiple traits, including resistance to pod borers and diseases. Exploitation of GP-3 and GP-4 has been attempted in soyabean, but it ended at the amphidiploid stage (Mignucci and Chamberlain, 1978). The novel sources of biotic and abiotic stresses from the perennial species of the subgenus, *Glycine* (GP-3) have not yet been fully exploited. *A. cardenasii* has probably been one of the most widely used sources of useful genes in groundnut to date, even if crosses involving other species have also been used.

The wild lentil taxa are potential reservoirs of valuable genes controlling several traits. To increase and sustain lentil production and productivity, new gene sources need to be identified and transferred from different GPs. To maximize and sustain common bean production, high-yielding, superior quality cultivars that are less dependent on water, fertilizer, and pesticides should be developed. A tiered breeding approach involving alien gene introgression, gene deployment, and multi-trait improvement programmes would be the most appropriate strategy to accomplish these goals. Cross incompatibilities are the major barriers to improve the productivity of faba bean. However, modern breeding tools succeeded in transferring genes across incompatible species and opened opportunities to develop high-yielding faba bean with reduced anti-nutritional factors. Marker-based assessment of genetic diversity in geographically specified germplasm sets of cultivated lupin species might speed up the process of assigning different genotypes to “Old World” and “New World” farmed species. Transgressive segregants identified in distant hybridisation programmes enable the development of high yielding pea cultivars.

Exploitation of wild *Vigna* species helps to discover novel alleles for yield improvement in different *Vigna* crops. The application of genomic tools to harness genetic diversity from wild relatives of cowpea has yet to be accessed. Introducing molecular genetics tools into cowpea distant hybridization helps in bypassing the incompatibility barriers and introgressing desirable alleles or haplotypes. Horse gram and rice bean are the climate-resilient future crops for dryland areas. Present and future challenges in the breeding of forage legumes demand for the exploration of genetic variation in breeding programmes. The most dependable and desirable way to use their wild relatives is to introgress desirable traits into modern cultivars. Forage yield, quality, and persistence are the most important economic traits and have been shown to be good candidates for the future forage breeding.

In conclusion, concerted integrated strategies should be used to identify and introgress the valuable genes housed in the CWRs of legume crops. This will provide us with a generation of a multitude of novel pre-bred lines that can be utilised in mainstream breeding to fight today’s climatic vagaries.

## Author contributions

IS, PS, GS, HA and SG conceived the idea. Authors have contributed in writing individual crops: GS (Pigeonpea), AD (Groundnut), PU (Chickpea), PKS (Lentil), GN (Moth bean and Rice bean), LG (Forage legumes), DK (Soybean), PK (Lupin), AK (Common bean), AT (Black gram), SS (Green gram), GK (Faba bean), MDP (Pea and Cowpea), and PH (Horse gram). SG and GS prepared the figures. HA, SG, RK, SK and AD collected the data and finalized the tables. HA, PS, GS, and SG compiled and edited the manuscript. IS supervised the study and finalized the manuscript. All authors contributed to the article and approved the submitted version.

## Acknowledgments

The authors would like to thank everyone who contributed to improving the quality of the review article at various stages of writing manuscript.

## Conflict of interest

The authors declare that the research was conducted in the absence of any commercial or financial relationships that could be construed as a potential conflict of interest.

## Publisher’s note

All claims expressed in this article are solely those of the authors and do not necessarily represent those of their affiliated organizations, or those of the publisher, the editors and the reviewers. Any product that may be evaluated in this article, or claim that may be made by its manufacturer, is not guaranteed or endorsed by the publisher.
